# Heat capacity anomalies of the molecular crystal 1-fluoro-adamantane at low temperatures

**DOI:** 10.1038/s41598-021-97973-2

**Published:** 2021-09-20

**Authors:** Daria Szewczyk, Jonathan F. Gebbia, Andrzej Jeżowski, Alexander I. Krivchikov, Tatiana Guidi, Claudio Cazorla, Josep-Lluís Tamarit

**Affiliations:** 1 Institute of Low Temperature and Structure Research PAS, Okólna 2, 50-422 Wrocław, Poland; 2 Grup de Caracterització de Materials, Departament de Física, EEBE and Research Center in Multiscale Science and Engineering, Universitat Politècnica de Catalunya, Eduard Maristany, 10-14, 08019 Barcelona, Catalonia Spain; 3 B. Verkin Institute for Low Temperature Physics and Engineering, NAS of Ukraine, Nauki Ave. 47, Kharkiv, 61103 Ukraine; 4 ISIS Facility, Rutherford Appleton Laboratory, Chilton, Didcot, OX11 0QX Oxfordshire UK; 5 Departament de Física, Universitat Politècnica de Catalunya, Campus Nord B4-B5, 08034 Barcelona, Spain

**Keywords:** Condensed-matter physics, Statistical physics, thermodynamics and nonlinear dynamics

## Abstract

Disorder–disorder phase transitions are rare in nature. Here, we present a comprehensive low-temperature experimental and theoretical study of the heat capacity and vibrational density of states of 1-fluoro-adamantane (C_10_H_15_F), an intriguing molecular crystal that presents a continuous disorder–disorder phase transition at *T* = 180 K and a low-temperature tetragonal phase that exhibits fractional fluorine occupancy. It is shown that fluorine occupancy disorder in the low-*T* phase of 1-fluoro-adamantane gives rise to the appearance of low-temperature glassy features in the corresponding specific heat (i.e., “boson peak” -BP-) and vibrational density of states. We identify the inflation of low-energy optical modes as the main responsible for the appearance of such glassy heat-capacity features and propose a straightforward correlation between the first localized optical mode and maximum BP temperature for disordered molecular crystals (either occupational or orientational). Thus, the present study provides new physical insights into the possible origins of the BP appearing in disordered materials and expands the set of molecular crystals in which “glassy-like” heat-capacity features have been observed.

## Introduction

Phase transitions between stable (or metastable) phases occur as a result of changes in the external conditions (e.g., temperature, pressure, magnetic or electric field, and/or mechanical stress)^1^. When a field-driven phase transition takes place, some physical properties of the material change either discontinuously (i.e., first-order phase transitions) or continuously (i.e., continuous or high-order phase transitions), and a specific thermodynamic potential (e.g., the Gibbs free energy, *G*) reflects that change. For instance, typical phase transitions entailing an abrupt change in volume (*V*) and entropy (*S*) are described by discontinuities in the first-derivative of the free energy (e.g., *S* = − ∂*G*/∂*T*). Consequently, response functions (i.e., second-derivatives of the free energy) like the thermal expansion, isothermal compressibility and heat capacity (*C*) present singularities at the phase transition points (e.g., *C*/*T* = ∂*S*/∂*T* = − ∂^2^*G*/∂*T*^2^). On the other hand, high-order phase transitions are characterized by discontinuities in the second or higher order derivatives of the free energy hence their response functions are discontinuous at the phase transition points. This is the typical case of order–disorder phase transitions^2,3^, in which the involved phases show no apparent volume and entropy differences at the transition points, in contrast to first-order phase transitions. A particular class of continuous phase transition that is quite rare are solid–solid disorder-disorder phase transitions^4^.

**Figure 1:**
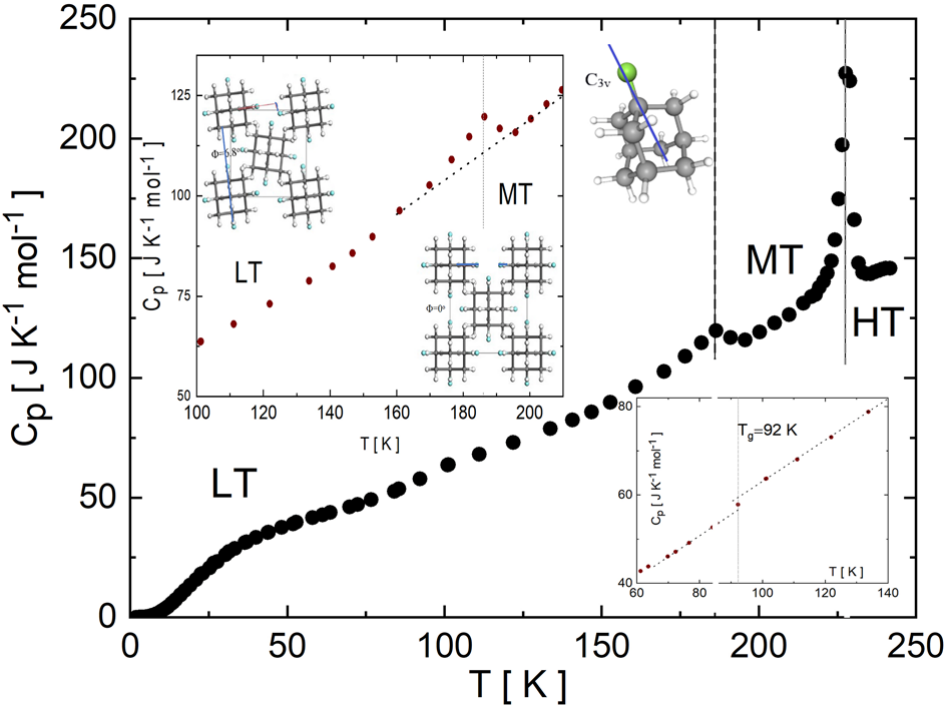
Molar﻿ heat capacity (*C*_*p*_) of 1-fluoro-adamantane expressed as a function of temperature. Left-top inset corresponds to the heat capacity data obtained for the temperature range 100–210 K with representations of the LT and MT phases. The angle between the C–F bond in the four equilibrium positions and the crystallographic tetragonal **a** (or **b**) axis, ϕ. Right-bottom inset magnifies the effect of the glass transition at 92 K. 1-fluoro-adamantane molecule with the *C*_*3v*_ point group symmetry is also enclosed.

Here, we investigate *T*-driven phase transitions in the molecular crystal 1-fluoro-adamantane (1F-A hereinafter) and the accompanying heat capacity behavior at low temperatures. Adamantane (C_10_H_16_) is a hydrocarbon formed by three or more interwoven cyclohexane rings that render *T*_*d*_ point group symmetry (see inset Fig. 1). Substitutions at either four of the tertiary carbons (1-) or six of the secondary carbons (2-) of adamantane give rise to the 1-X-adamantane (with *C*_*3v*_ point group symmetry) or 2-X-adamantane derivatives (with *C*_*s*_ symmetry). All 1-X- and 2-X-adamantane species display rich polymorphism and a large number of experimental and theoretical investigations have been devoted to their study^5–9^. A common characteristic of 1-X- and 2-X-adamantane derivatives is that they form orientationally disordered (OD) crystals, in which translationally ordered molecules perform reorientational jumps over well-defined orientations^4,10–12^.

Upon decreasing temperature, the reorientational dynamics slows down and either the OD phase transforms to a lower symmetry phase (entailing reduction of orientational disorder) or the system reaches a non-ergodic state (orientational glass, OG or glassy crystal). In general, the path from the OD to a fully ordered phase (if reached) is sequenced by several phase transitions, each one entailing a decrease in the degree of order and, mandatorily, a reduction of the lattice symmetry. In some cases, due to the low-thermal available energy, complete order is never achieved and consequently some orientational or occupational disorder remains in the crystal. On further cooling, such a disorder is retained and a non-ergodic state is attained^12–14^. 1F-A belongs to the class of molecular crystals that can stabilize a non-ergodic state at very low temperatures^11,15^.

On cooling from the liquid state, 1F-A crystallizes at ca. 525 K in an OD cubic phase (space group *Fm*$$\overline{3}$$*m*, Z = 4)^16,17^. This OD high-temperature (HT) phase is stable down to ca. 225 K and has been fully characterized by several experimental techniques^11,17–21^. On further cooling, the HT phase transforms to an intermediate (MT) tetragonal phase with space group *P*4_2_/nmc (Z = 2). This phase transition entails well-defined entropy and volume discontinuities, clearly revealing its first-order character. The MT tetragonal phase was considered to be the stable one all the way down to very low temperatures (~ 1–10 K), however, recent second harmonic generation measurements^15^ have evidenced a group-subgroup continuous phase transition occurring at ca. 180 K from the MT phase to another low-temperature (LT) tetragonal phase (space group $$P\overline{4}2_{1}$$$$c$$, Z = 2). The OD HT phase displays eight equilibrium positions for the fluorine atom (with equal occupancy factors) whereas in both MT and LT phases such occupational disorder reduces to four equivalent F sites (with equal occupancy factors, 1/4). Thus, disorder in the tetragonal MT and LT phases consists of large rotational jumps over equivalent F sites located around the corresponding two- and three-fold symmetry axes, respectively. The physical picture of the continuous MT to LT phase transition corresponds to an alignment of the C–F bond with the relevant **a** (or **b**) tetragonal axis (see Fig. 1). At 90 K, for instance, the C–F bond angle (which corresponds to the three-fold axis of the *C*_*3v*_ molecule and thus to the molecular dipole direction) is found to be 6.8° whereas at ca. 180 K is 0° (i.e., upon increasing *T* two new mirror planes perpendicular to the **a** and **b** tetragonal axes, appear thus increasing the lattice symmetry—see inset in Fig. 1). In fact, the high-order nature of this disorder-disorder phase transition has been corroborated by the continuity of the relaxation time measured in dielectric spectroscopy experiments^11^.

In this work, we report measurements for the specific heat capacity (*C*_p_) of 1F-A during the continuous MT to LT phase transition and disclose intriguing *C*_p_ anomalies for the corresponding LT phase. In particular, the existence of a boson peak (BP), which is characteristic of orientational and structural glasses, is clearly demonstrated. Meanwhile, the low-*T* specific heat capacity upturn appearing in the *C*_*p*_*/T*^3^ representation of two-level quantum tunneling systems^22–24^ is found to be absent. Furthermore, we compare the BP characteristics of different adamantane derivatives displaying either occupational disordered phases (e.g., 2-adamantanone) or orientationally disordered phases (e.g., 1-cyano-adamantane)^9,12,25^ and propose a general and direct correlation between their first localized optical mode and maximum BP temperature. First-principles simulations based on density functional theory (DFT) complement our experimental study by providing further characterization of the vibrational properties of 1F-A. The present study, therefore, provides new physical insights into the possible origins of the BP appearing in disordered materials and enlarges the spectrum of molecular crystals in which “glassy-like” *C*_p_ features have been observed.

### Experimental and computational details

Commercially available 1-fluoro-adamantane was purchased from ABCR (99% purity), and was used without further treatment. A pellet was built up by pressing the provided powder sample using a hydraulic press (1500 kg cm^−2^) through a specially designed container.

Heat capacity as a function of temperature was determined by means of a Physical Property Measurement System (PPMS) from Quantum Design Inc. operated in the heat capacity mode. The mass of the sample loaded into the calorimeter was 9.4 mg, and the molecular mass calculated from the stoichiometry was 154.22 g mol^−1^. Heat capacity measurements were carried out in a temperature range 1.8–250 K with an accuracy better than 1%.

We performed Inelastic Neutron Scattering (INS) measurements using the direct geometry spectrometer MARI. The sample (m ≈ 2–3 g) was loaded into an aluminium sample holder with annular geometry designed for powder samples. The INS data were collected at a temperature of 5 K with an incident energy of 15 meV using a Gd Fermi chopper spinning at 200 Hz.

The experimental data were treated using Mantid software framework^26^ with a standard method to obtain the inelastic spectrum *S*(*Q*,ω). The neutron weighted vibrational density of states (VDOS) as a function of the energy transferred have been computed using the *ComputeIncoherentDOS* Mantid algorithm.

Density functional theory (DFT) calculations^27^ were performed based on the PBEsol functional^28^ with the VASP software^29^. Long-ranged dispersion interactions in the system were captured with Grimme’s DFT-D3 method^30^. Wave functions were represented in a plane-wave basis truncated at 650 eV and a Monkhorst–Pack **k**-point grid of 4 × 4 × 3 was employed for integrations within the Brillouin zone. Geometry relaxations were performed for optimizing the lattice vectors of bulk 1F-A at zero temperature. Four different F occupancy unit cell arrangements were considered to somehow reproduce 1F-A occupancy disorder. To estimate the harmonic density of vibrational states at the center of the Brillouin zone (i.e., Γ reciprocal space point) we employed density functional perturbation theory^29^. To estimate *g*(*ω*) by fully taking into account the anharmonicity of the system, we calculated the Fourier transform of the velocity–velocity autocorrelation function^31^ obtained from a long ab initio molecular dynamics (AIMD) run (~ 100 ps) performed at a fixed temperature of 100 K and the equilibrium volume. The supercell employed in the AIMD simulations contained 432 atoms.

## Results and discussion

The molar heat capacity (*C*_*p*_) measured for 1-fluoro-adamantane within the temperature range 1.8–250 K (upon heating) is shown in Fig. 1. In addition to the first-order MT → HT phase transition occurring at 227 K and that corresponds to the *C*_*p*_ maximum, several other transformations are also identified. A small but clear *C*_p_ discontinuity signaling the continuous LT to MT phase transition extends from 170 to 195 K and presents a maximum (ca. 7.5 J mol^−1^ K^−1^) at around 186 K (see left-top inset in Fig. 1).

The shape of the heat capacity curve close to the LT to MT phase transition appears to be quite symmetrical at both sides of the critical temperature. This feature differs significantly from the exponential increase (singularity) observed for first-order order–disorder phase transitions, and corroborates the continuous nature of the LT to MT phase transition. In fact, a critical exponent (β) can be estimated for the temperature variation of the order-parameter, $\propto {(T-{T}_{c})}^{-\beta }$, around the LT to MT transition point, defined either as the angle ϕ (see inset in Fig. 1) formed by the C–F bond of the four F equilibrium positions and **a** (or **b**) tetragonal axis, or as the intensity of the second harmonic signal (which vanishes at the centrosymmetric MT phase)^15^.

For the disordered LT phase, it was found by broadband dielectric spectroscopy that the molecular reorientational jumps freeze at around 92 K^11^, i.e., the temperature at which the relaxation time reaches a value of 100 s (defined as the glass transition temperature regardless the kind of frozen disorder). Figure 1 (see right-bottom inset) shows a small, but measurable, heat capacity discontinuity at around 90 K, which is a consequence of the freezing of the degrees of freedom involved in F occupational disorder.

The most characteristic *C*_p_ fingerprints of glassy systems at low temperatures are: (i) a (non-electronic) linear contribution (*C*_*p*_ ∝ *T*) stemming from “two-level system” (TLS) quantum tunneling processes, and (ii) a significant departure from the Debye law *C*_*p*_ ∝ *T*^3^ (or, equivalently, from *g*(*ω*) ∝ *ω*^2^, where *g*(*ω*) represents the density of vibrational states—VDOS) due to an excess of low-energy vibrational states. Thus, in the low-temperature limit in which only low-energy phonons are excited, the most general approximation for the specific heat as a function of temperature can be written as^32^:

1 $$C_{p} \left( T \right) = C_{TLS} \cdot T + C_{D} \cdot (T/{\theta}_{D} )^{{3}} + C_{SPM} \cdot T^{{5}} ,$$

where the *C*_*TLS*_ term accounts for TLS effects^23,24^, ${C}_{D}=\frac{12}{5}{\pi }^{4}R$ (*R* is the universal gas constant) is the Debye contribution characterized by the Debye temperature, θ_*D*_, and *C*_*SPM*_ represents an excess of quasi-localized lattice vibrations (called “soft modes”)^33,34^.

**Figure 2:**
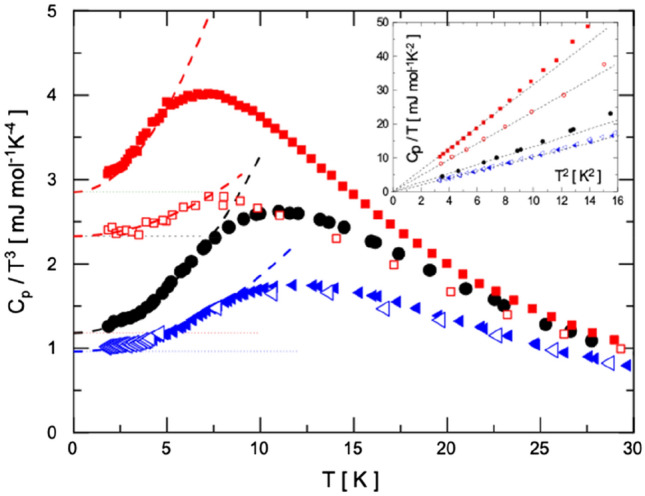
Debye-reduced specific heat capacity data, *C*_*p*_*/T*^3^, for the LT phase of 1F-A (black circles) as well as for some adamantane derivatives presenting ordered (empty symbols) and disordered (full symbols) phases: cyano-adamantane (red squares) and 2-adamantanone (blue triangles). Inset: Specific heat capacity data plotted as *C*_*p*_/*T* vs. *T*^2^ at low temperature (from 2 to 4 K).

**Figure 3:**
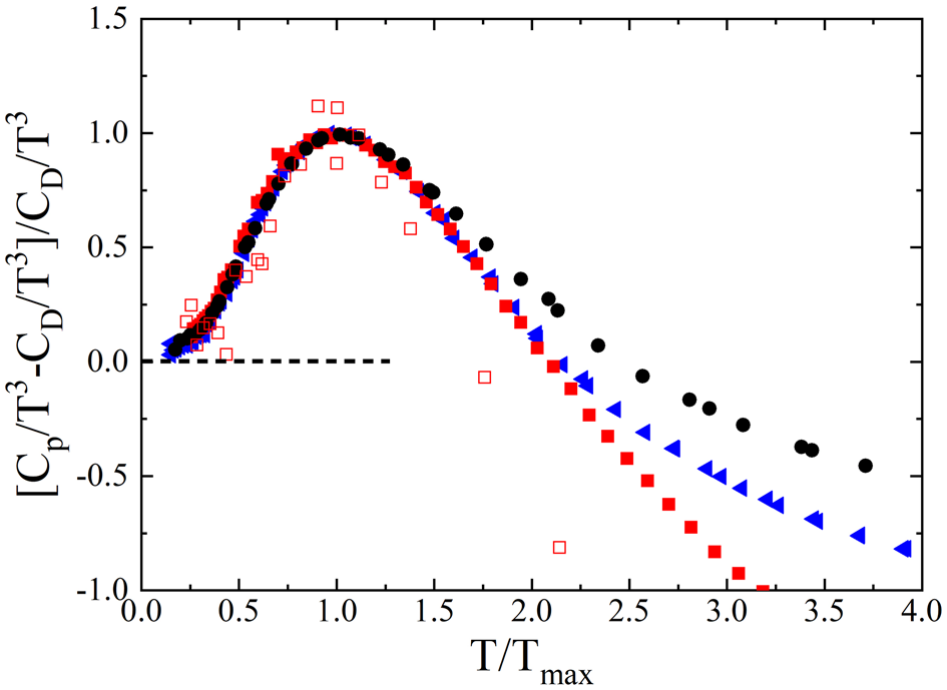
Specific﻿ heat capacity difference taken with respect to and normalized by the Debye contribution (*C*_*D*_ values) and expressed as a function of the reduced temperature *T/T*_*max*_ (*T*_*max*_ being the maximum of the boson peak as determined from the *C*_*p*_*/T*^3^ representation) for several adamantane derivatives presenting ordered (empty symbols) and disordered (full symbols) phases: cyano-adamantane (red squares), 2-adamantanone (blue triangles) and 1F-A (black circles).

Figure 2 shows the Debye-reduced specific heat data, *C*_p_/*T*^3^, measured for the LT phase of 1F-A at low temperatures. The appearance of a boson peak (BP) at *T*_*max*_ ≈ 10.8 K and the absence of TLS regime are evidenced therein. (The inset in Fig. 2 further demonstrates that the linear contribution to *C*_p_ is zero, *C*_*TLS*_ = 0, via a numerical fit of the *C*_*p*_/*T* function to *T*^2^^35^). The clear *C*_*p*_/*T*^3^ departure from a constant value starting at around 3–4 K, evidences the existence of low-energy vibrational soft-mode excitations. At temperatures below *T*_max_ = *T*_bp_, the specific heat data is necessarily accounted for by the additional term *C*_*SPM*_ (Fig. 3). Table 1 encloses the results of the numerical specific-heat fits performed according to Eq. (1) for 1F-A as well as for other adamantane derivatives (vide infra).

**Table 1:** Low-temperature specific heat capacity parameters according to Eq. (1) (*θ*_*D*_, *C*_*5*_), temperature of the maximum of the boson (*T*_bp_) and energy of the lowest energy mode determined from Raman (*E*_0_).

Material/phase (O/D)	*θ*_D_ (K)	*C*_5_ (mJ mol^−1^ K^−6^)	*T*_bp_ (K)	*E*_b_ = 5*T*_bp_ (K)	*E*_0_ (cm^−1^)
C_10_H_15_F/LT (D)	118.1 ± 0.5	0.021	10.8	54	30^c^
C_10_H_14_O/Monoclinic (D)	124.4^a^	0.0086^a^	12.2^a^	61	36^d^
C_10_H_14_O Orthorhombic (O)	124.4^a^	0.010^a^	12.2^a^	61	42^d^
C_10_H_15_CN Ordered (O)	93.0^b^	0.0090^b^	8.0^b^	38	–
C_10_H_15_CN fcc (D)	87.0^b^	0.040^b^	7.1^b^	35.5	26^e^

^a^Ref.^25^.

^b^Ref.^9^.

^c^Ref.^21^.

^d^Ref.^36^.

^e^Ref.^37^.

It is worth mentioning that the heat-capacity values reported in Table 1 include orientationally disordered phases (e.g., the plastic phase of cyano-adamantane), occupational disordered phases (e.g., the monoclinic phase of 2-adamantanone and the LT phase of 1F-A), as well as fully ordered phases (e.g., those for cyano-adamantane and the orthorhombic stable phase of 2-adamantanone). It is also noted that the Debye temperatures reported in Table 1 are not correlated with the ordering features of the involved phases (e.g., the θ_*D*_ values estimated for the ordered and disordered phases of 2-adamantanone are virtually the same). Such an experimental outcome reinforces the idea that for understanding the universal origins of the TLS and BP it is necessary to consider additional aspects besides the level of crystalline disorder^38^.

Recently, the possible relevance of the competition between acoustic and low-energy optical modes on the emergence of anomalous glassy-like *C*_p_ features in crystals has been discussed^14,38^. In particular, it has been proposed that hybridizations between propagating and quasi-localized diffusive excitations, leading to the piling up of low-energy optical phonons that interact with the propagating acoustic modes, might be the responsible for the appearance of the BP anomaly (independently of the level of disorder of the involved phases)^39,40^. A novel theory has been developed based on such a proposal^41,42^, in which the main contribution to the BP is assumed to come from the lowest energy optical mode at the Brillouin zone center, ω_o_, which can interact with propagating acoustic modes^43,44^. Within this theoretical framework, the energy of the first (lowest energy) optical mode should be correlated with the appearance of the BP at *T*_max_ = *T*_bp_.

**Figure 4:**
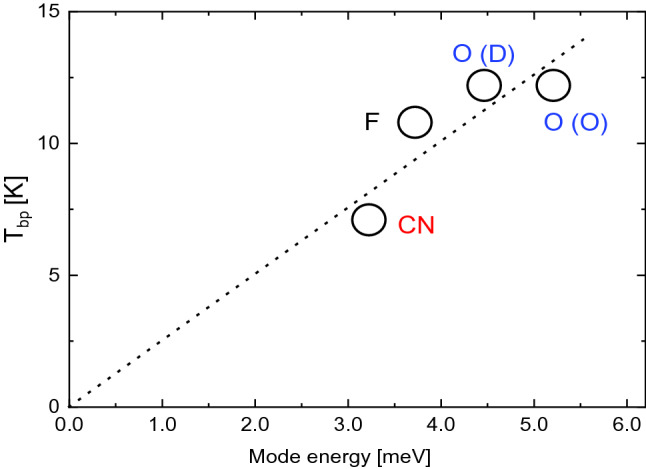
Temperature of the boson peak, obtained as the maximum of *C*_*p*_*/T*^3^, expressed as a function of the energy of the first (low-energy) optic mode for 1F-A (F), orthorhombic ordered (O) and monoclinic disordered (D) phases of 2-adamantanone (O) and the plastic phase of 1-cyano-adamantane (CN). The dotted line represents a linear fit to the plotted data.

Figure 4 shows the maximum BP temperature determined from experimental *C*_*p*_*/T*^3^ measurements and represented as a function of the corresponding lowest-energy optical mode, *E*_0_, obtained from Raman spectroscopy data reported in the literature (Table 1). Interestingly, it turns out that *T*_bp_ appears to depend almost linearly on *E*_0_. This incipient *T*_bp_–*E*_0_ correlation suggests that, first, the appearance of BP in translationally ordered systems responds to the pilling up of low-energy optical modes and, second, interactions between quasi-localized optical and well-defined acoustic modes near the Brillouin-zone boundaries are possible. Moreover, such an inference is quite general as it does not discriminate between different types of disorder (i.e., orientational for the case of cyano-adamantane and occupational for the monoclinic phase of 2-adamantanone and 1F-A), thus upholding similar conclusions reported in previous works^14^.

**Figure 5:**
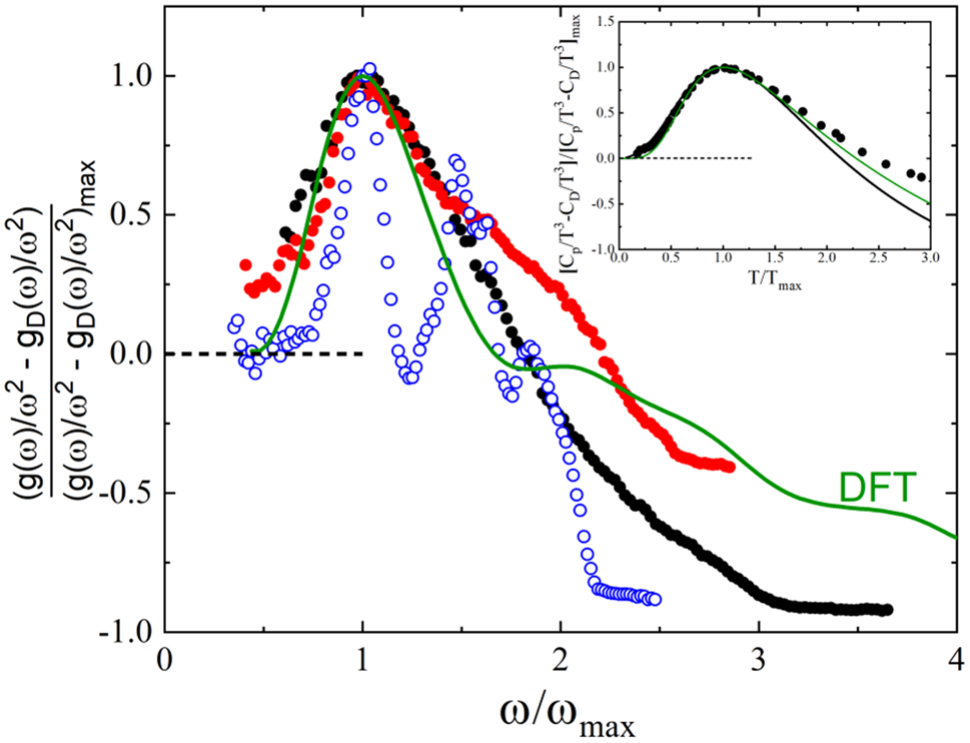
Normalized vibrational density of states in the reduced form (*g*(ω)/ω^2^) expressed as a function of ω when scaled by its maximum (i.e., ω/ω_max_) for 1F-A (red circles) as well as for the occupational disordered (full black circles) and the ordered stable (empty blue circles) 2-adamantanone phases. Theoretical DFT results are obtained for 1F-A. Inset: Experimental (black line) and calculated (green line) specific heat capacity of 1F-A obtained from the corresponding VDOS. Black circles represent the heat capacity data measured directly in the experiments. The *C*_*p*_ data are normalized to the corresponding Debye contribution (*C*_*D*_ values) and expressed as a function of the reduced temperature *T*/*T*_max_.

An interesting feature shown in Fig. 3 is the similarity among the Debye-scaled low-temperature specific heat of the considered adamantane crystals when represented as a function of the *T*_bp_-scaled temperature. Although the corresponding phonon dispersion curves are not available, it can be hypothesized based on their molecular correspondence that the energy of the lowest-energy optical modes is quite similar for all them (even though the translational-rotational coupling of the low-energy localized optical modes with acoustic propagating phonons may differ). Such a hypothesis is actually valid for 2-adamantanone, as it has been experimentally demonstrated for its monoclinic phase through analysis of its vibrational eigenvectors^45^. Within the same line of reasoning, the similarity between the vibrational density of states (VDOS), *g*(ω)*,* of some of the aforementioned compounds is also evidenced when the *g*(ω)/ω^2^ function is normalized to its maximum value and represented as a function of ω/ω_*max*_, where ω_*max*_ is the frequency at which the *g*(ω)/ω^2^ maximum appears (see Fig. 5). Despite that the experimental VDOS have been measured within a reduced energy range, the calculation of the specific heat capacity from such *g*(ω), by adjusting the Debye level, matches very well the corresponding *C*_p_ experimental values (see inset in Fig. 5). Such an agreement illustrates that, as far as for the low-temperature contributions to the specific heat capacity, only the lowest-energy modes are relevant to explain the appearance of the BP.

Figure 5 also includes the VDOS calculated from ab initio molecular dynamics (AIMD) simulations for the LT phase of 1F-A, which are based on density functional theory calculations (section “Experimental and computational details”). The theoretical VDOS in fact provides further evidence for the pilling up of low-energy modes, as shown by a clear *g*(ω)/ω^2^ maximum appearing at a frequency of ω^*AIMD*^_*max*_ ≈ 3 meV. Moreover, the lowest-energy (average) optical mode estimated for 1F-A at the center of the Brillouin zone by neglecting temperature effects and anharmonicity is ≈ 4 ± 1 meV, which is in consistent agreement with ω^*AIMD*^_*max*_. It is worth noting that the specific heat capacity obtained from the calculated VDOS (see inset in Fig. 5) agrees remarkably well with the experimental *C*_p_ values and reproduces also the heat capacity BP anomaly (as well as the absence of TLS regime). Thus, the theoretical VDOS results obtained for 1F-A support the proposition that the existence of low-energy modes may account for the appearance of the BP heat capacity anomaly at low temperatures.

## Conclusions

Specific heat capacity measurements are presented for the molecular crystal 1-fluoro-adamantane over the broad temperature interval 2 ≤ *T* ≤ 250 K. A high-order disorder-disorder phase transition between two tetragonal phases is identified at around 186 K, thus confirming the symmetry-broken (group-subgroup) phase transition previously detected from second harmonic generation measurements. The low-temperature phase (LT) involved in the phase transformation evolves into a non-ergodic phase in which occupational F disorder is frozen at temperatures below ca. 90 K.

The disordered character of the LT phase induces the appearance of glassy *C*_*p*_ features commonly found in structural (canonical) and orientational glasses, like the boson peak in the *C*_*p*_/*T*^3^ versus *T* representation. Such anomalous *C*_*p*_ features originated by F occupational disorder are very similar to those previously found in systems presenting occupational and orientational disorder (i.e., 2-adamantanone^25,45^ and cyano-adamantane, respectively). Moreover, regardless the type of disorder, the maximum temperature of the boson peak appears to be directly correlated with the energy of the first optical mode (“soft-mode”) appearing at the center of the Brillouin zone of the crystal. Therefore, the reported results suggest that, at least for translationally ordered crystals, the origin of the boson peak is linked to the ability of the system to couple acoustic (propagating) and optic (quasi-localized) phonon modes. Thus, as far as for the low-temperature contributions to the specific heat capacity, only the lowest-energy vibrational excitations are relevant to explain the appearance of the BP.
